# Supplementary material for: Assessing the value of integrating national longitudinal shopping data into respiratory disease forecasting models
Source: Nat Commun. 2023 Nov 21;14:7258. doi: 10.1038/s41467-023-42776-4 (PMC10663456; doi:10.1038/s41467-023-42776-4)
Supplement: Supplementary file 1 — Supplementary Information [file 41467_2023_42776_MOESM1_ESM.pdf]

Assessing the value of integrating national longitudinal shopping data into respiratory disease forecasting models

Supplementary Documents

Contents

Supplementary Figures

1. Supplementary Fig. 1
   Scatter plots with line of best fit showing positive correlation between sales of cough, decongestant and throat medications, and deaths from respiratory disease.
2. Supplementary Fig. 2
   Diagram illustrating forecast horizons and timings of weekly sales variable inputs.
3. Supplement Fig. 3
   Line Plot of PADRUS and PADRUNOS predictions with standard error.
4. Supplement Fig. 4
   Point plots illustrating differences in RMSE, MAE and R-squared between the models PADRUS and PADRUNOS across different monthly results.

Supplementary Tables

5. Supplementary Table 1
   MCR Permutation Feature Importance Bounds.
6. Supplementary Table 2
   Data used in the models for the target for predictions and feature creation.
7. Supplementary Table 3
   Results from experimental models predicting registered deaths from respiratory disease.
8. Supplementary Table 4
   Weekly prediction scores for PADRUS and PADRUNOS models.
9. Supplementary Table 5
   Prediction scores by Lower Tier Local Authorities (LTLA) for PADRUS.

References

10. References (relevant to Supplementary Table 2)

Supplementary Fig. 1.

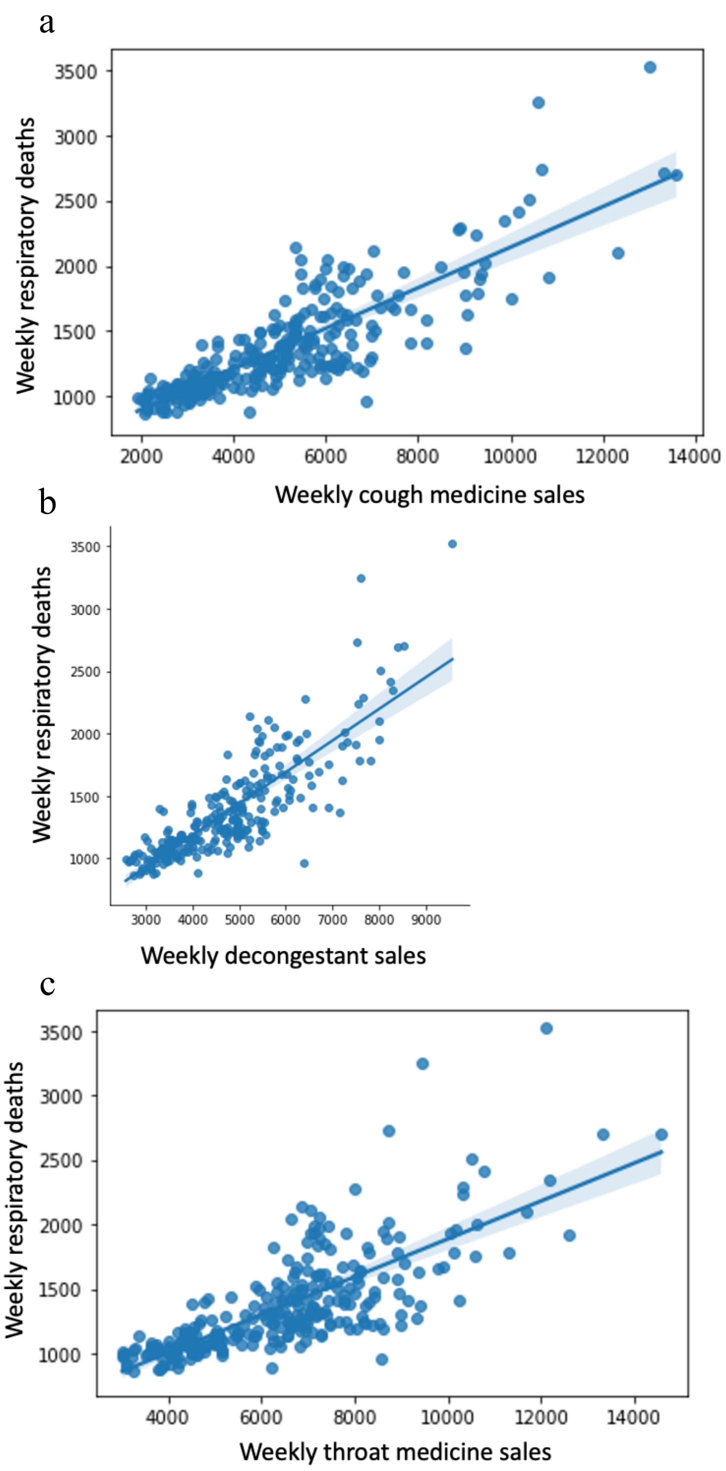

Supplementary Fig. 1. Scatter plots with line of best fit showing positive correlation between sales of a) cough, b) decongestant and c) throat medications, and deaths from respiratory disease. Weekly national deaths and sales for England and Wales from November 2009 and April 2015. Sales data from a sample of loyalty card data transactions. Mortality data from Office of National Statistics. There is a forecast horizon of 17 days between weekly sales and weekly deaths.

Supplementary Fig. 2

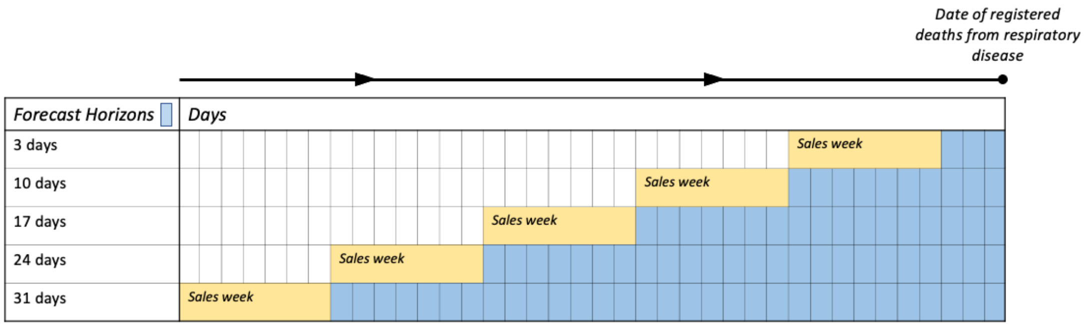

Supplemtary Fig 2. Diagram illustrating forecast horizons and timings of weekly sales variable inputs

Supplementary Fig. 3.

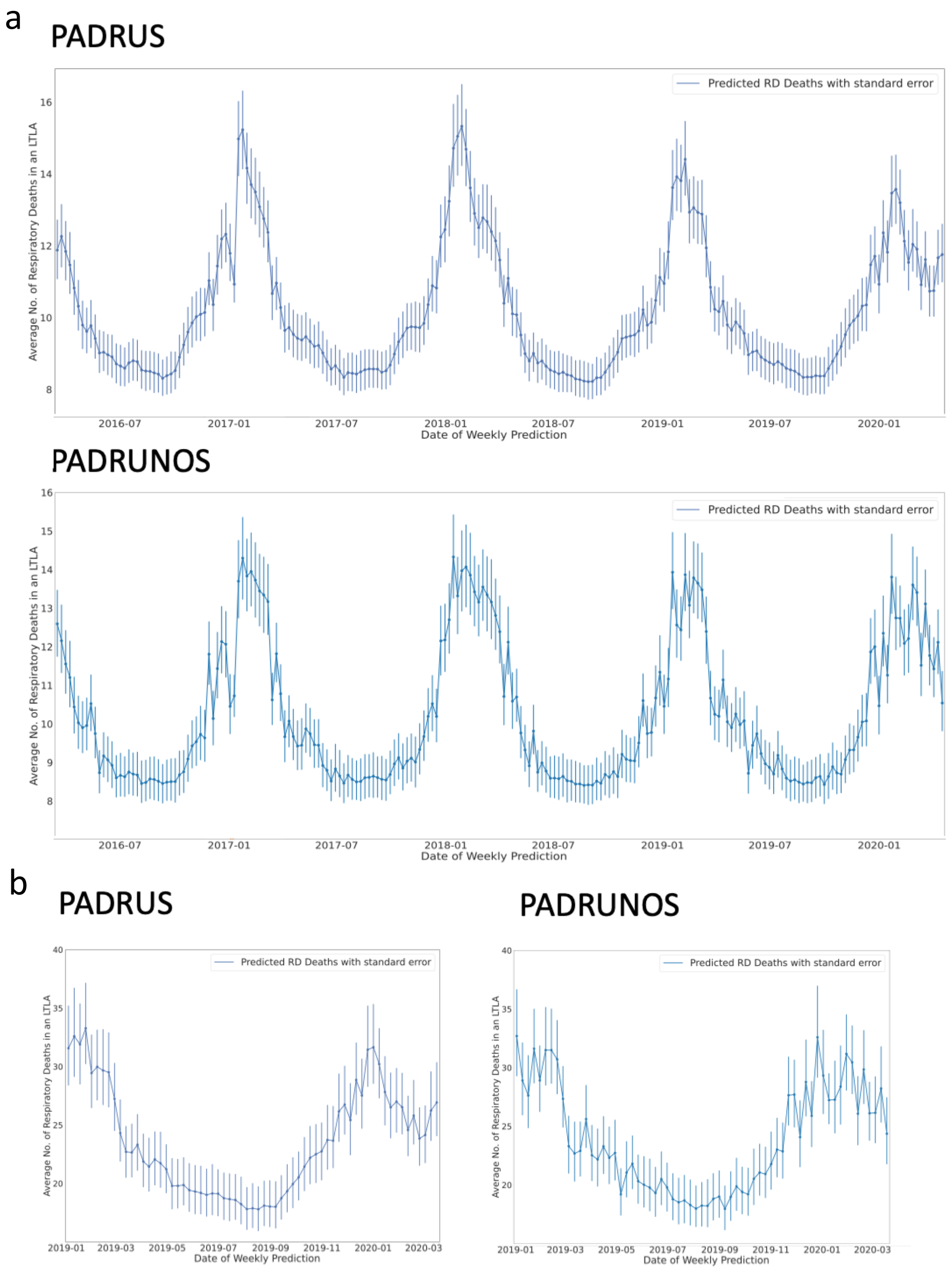

Supplementary Fig. 3. Line Plot of PADRUS and PADRUNOS predictions of respiratory deaths with standard error. Results are from 66,254 datapoints (45,844 training datapoints, 20,410 testing datapoints) of 211 weekly predictions of deaths from respiratory disease for 314 English Local Authorities from 18th March 2016 to 27th March 2020. **b** panels provide PADRUS and PADRUNOS predictions for 41 LTLA areas without suppression with standard error on the test data time period only

Supplementary Fig. 4

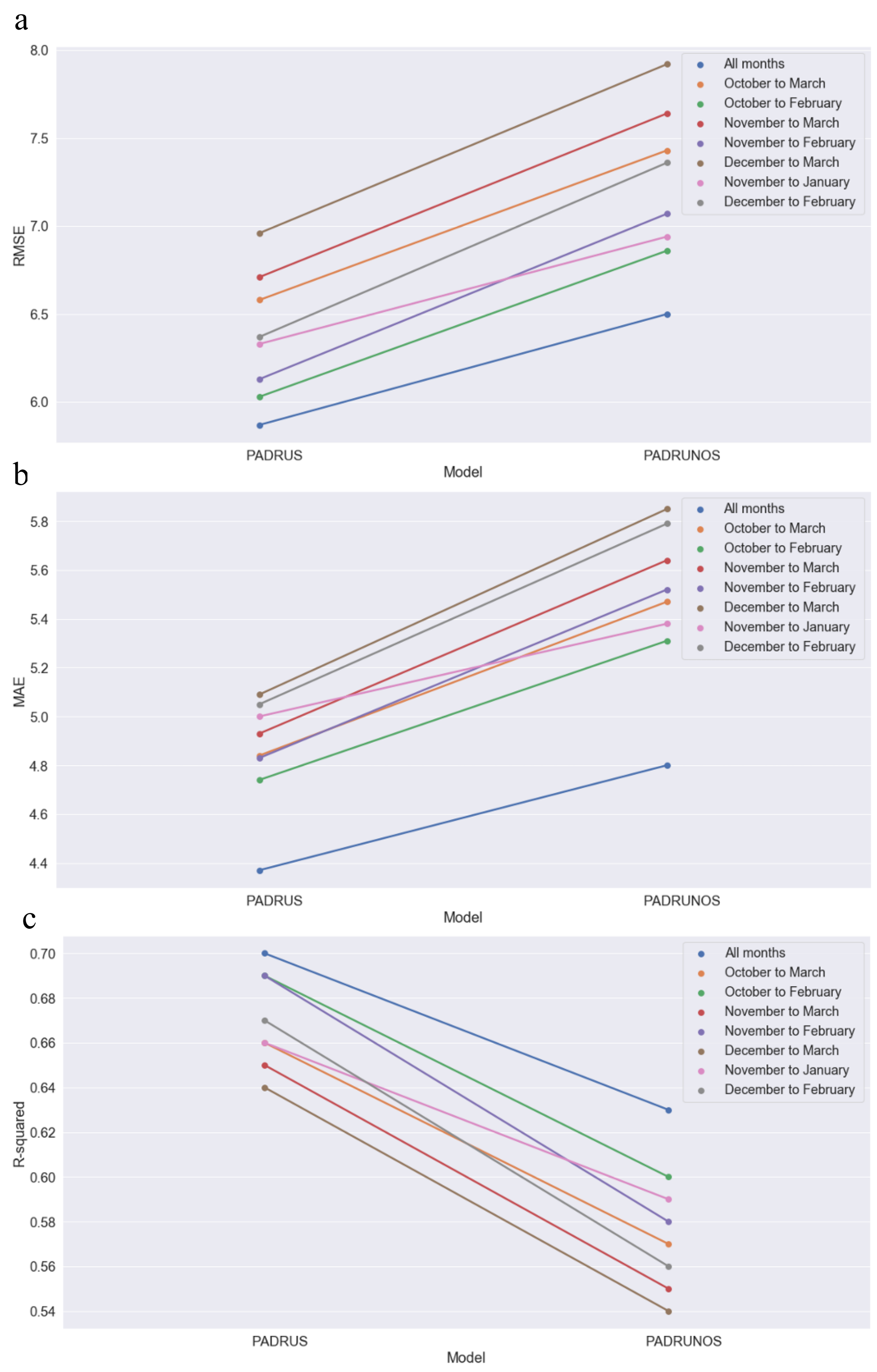

Supplementary Fig. 4. Point plots illustrating differences in a) RMSE, b) MAE and c) R-squared between the models PADRUS and PADRUNOS across different monthly results. Models predict weekly deaths in English Local Authorities 18/03/2016 to 27/03/2020 with a forecast horizon of 17 days.

Supplementary Table 1

Supplementary Table 1. MCR Permutation Feature Importance Bounds for PADRUS and Permutation Feature Importance Scores for an arbitrary instance (PIA) of PADRUS.

Variable Feature Name	MCR-	MCR- rank	MCR+	MCR+ rank	PIA	PIA rank
Population over65	3.95	1	10.75	1	0.0775	1
Population 50to64	2.97	2	10.03	2	0.0591	2
Population 25to49	2.57	3	6.08	3	0.0408	3
Population 16to24	1.23	4	4.27	4	0.0231	4
Weekly cough medications 24 days in advance_24	0.93	5	1.18	18	0.0196	5
Weekly dry cough medications 24 days in advance	0.93	6	1.19	17	0.0178	6
Weekly dry cough medications 17 days in advance	0.77	7	1.11	20	0.0139	8
Weekly cough medications 17 days in advance	0.72	8	1.06	22	0.0142	7
Index of multiple deprivation concentration	0.71	9	1.58	5	0.0127	9
Weekly decongestant medications 17 days in advance	0.69	10	1.04	23	0.0101	13
Weekly minimum temperature 17 days in advance	0.64	11	1.03	24	0.0111	12
Weekly maximum temperature 17 days in advance	0.63	12	1.01	25	0.0113	11
Weekly average temperature 17 days in advance	0.58	13	1.00	26	0.0118	10

Continued on next page

Table 1 – continued from previous page

Variable Feature Name	MCR-	MCR- rank	MCR+	MCR+ rank	PIA	PIA Rank
Number of week in the year	0.56	14	0.73	39	0.0098	14
Weekly decongestant medications 24 days in advance	0.54	15	0.78	38	0.0097	15
Weekly throat medications 17 days in advance	0.39	16	0.64	43	0.0068	20
Weekly sales in LTLA 17 days in advance	0.39	17	0.78	37	0.0071	18
Percentage of semi-detached (housing)	0.38	18	1.2	15	0.0064	23
Weekly cough medications local ratios 17 days in advance	0.38	19	0.58	44	0.0069	19
Index of multiple deprivation extent	0.37	20	1.39	6	0.0064	22
Weekly throat medications 24 days in advance	0.36	21	0.53	48	0.0076	17
Weekly dry cough medications local ratios 17 days in advance	0.36	22	0.56	47	0.0067	21
Percentage of flats (housing)	0.32	23	1.29	13	0.0076	16
Weekly sales in LTLA 24 days in advance	0.32	24	0.64	42	0.0057	25
Barriers to Housing and Services Domain Score	0.29	25	1.38	8	0.0051	27
Weekly decongestant medications local ratios 17 days in advance	0.28	26	0.43	49	0.0051	28
Index of multiple deprivation score	0.28	27	1.2	16	0.0063	24
Index of multiple deprivation rank	0.27	28	1.23	14	0.0051	26
Living Environment Deprivation Domain IMD Score	0.23	29	1.38	7	0.004	31
Weekly throat medications local ratios 17 days in advance	0.23	30	0.34	50	0.0042	30
Percentage of detached (housing)	0.22	31	1.1	21	0.0047	29
Percentage of residential land (land use)	0.22	32	1.37	9	0.0037	33
Crime Domain IMD Score	0.22	33	0.8	36	0.0037	32
Population density	0.2	34	1.36	10	0.0035	35
Percentage of agricultural (land use)	0.18	35	0.84	32	0.0028	41
Percentage of community buildings (land use)	0.18	36	0.98	28	0.0027	42
Weekly cough medications local multiplier 17 days in advance	0.18	37	0.33	51	0.0031	39
Percentage of pre-1983 (housing)	0.17	38	0.84	33	0.0033	37

Continued on next page

Table 1 – continued from previous page

Variable Feature Name	MCR-	MCR- rank	MCR+	MCR+ rank	PIA	PIA Rank
Percentage of people with non-white ethnicity	0.17	39	1.34	11	0.0036	34
Percentage of lone parents	0.17	40	0.86	31	0.0034	36
Percentage of natural (land use)	0.16	41	1.32	12	0.0033	38
Percentage of transport and utilities (land use)	0.16	42	0.99	27	0.0026	44
Weekly decongestant medications multiplier 17 days in advance	0.16	43	0.27	53	0.0029	40
Weekly dry cough medications multiplier 17 days in advance	0.15	44	0.28	52	0.0027	43
Percentage of pre-1940 (housing)	0.14	45	0.81	35	0.0026	45
Weekly throat medications multiplier 17 days in advance	0.13	46	0.2	54	0.0025	46
Percentage of industrial land (land use)	0.13	47	0.87	30	0.0022	49
Percentage of terraced (housing)	0.13	48	0.71	40	0.0024	48
Percentage of pre-1973 (housing)	0.13	49	0.84	34	0.0024	47
Percentage of pre-1919 (housing)	0.12	50	0.64	41	0.0019	52
Weekly average rainfall 17 days in advance	0.12	51	0.18	55	0.002	50
Weekly total rainfall 17 days in advance	0.1	52	0.18	56	0.002	51
Percentage of recreation (land use)	0.1	53	1.16	19	0.0018	54
Percentage of "other children" in families	0.1	54	0.89	29	0.0019	53
Percentage of females	0.08	55	0.57	45	0.0014	56
Percentage of males	0.07	56	0.57	46	0.0014	55

Supplementary Table 2

Supplementary Table 2. Table reporting data used in the models for the target for predictions and feature creation (variable inputs). (All inputs are aggregated to 314 LTLAs across England, and weekly rates if the variable is dynamic with a 17 day lag unless stated otherwise.

Data Description	Data Source	Research relating variable to respiratory disease	Target (y)	Temporality	Group for MCR
ONS (Office for National Statistics) deaths registered weekly in England from diseases of the respiratory system (ICD-10 Coding J00–J99)	NHSX/Digital receives data through request from ONS - https://digital.nhs.uk/services/primary-care-mortality-database.	N/A	Weekly deaths from respiratory disease in a LTLA	dynamic	N/A
-	-	-	Feature (X)	-	-
Week number	N/A	Moriyama,M., Hugentobler,W.J.,& Iwasaki, A. [5]	Number of week from 1 to 52	dynamic	Week
Weekly sales data from a UK high street retailer with stores distributed across the UK	University partnership with UK Retailer	Davies & Finch [1]	Total weekly sales	dynamic	Sales
			Weekly sales of decongestant	dynamic	Sales
			Weekly sales of throat meds	dynamic	sales
			Weekly sales of dry cough meds	dynamic	sales
			Weekly sales of all cough meds	dynamic	sales
			Total weekly sales 24 day lag	dynamic	sales
			Weekly sales of decongestant 24 day lag	dynamic	sales
			Weekly sales of throat meds 24 day lag	dynamic	sales

			Weekly sales of dry cough meds 24 day lag	dynamic	sales
			Weekly sales of all cough meds 24 day lag	dynamic	sales
			Local Ratio of Weekly sales of decongestant	dynamic	sales
			Local Ratio of Weekly sales of throat meds	dynamic	sales
			Local Ratio of Weekly sales of dry cough meds	dynamic	sales
			Local Ratio of Weekly sales of all cough meds	dynamic	sales
			Multiplier of Weekly sales of decongestant	dynamic	sales
			Multiplier of Weekly sales of throat meds	dynamic	sales
			Multiplier of Weekly sales of dry cough meds	dynamic	sales
			Multiplier of Weekly sales of all cough meds	dynamic	sales
English indices of deprivation 2019. Index of Multiple deprivation ranks Lower-layer Super Output Areas in England from 1 (most deprived area) to 32,844 (least deprived area). Combines data from seven domains: Income Deprivation (22.5%), Employment Deprivation (22.5%), Education, Skills and Training Deprivation (13.5%), Health Deprivation and Disability (13.5%), Crime (9.3%), Barriers to Housing and Services (9.3%), Living Environment Deprivation (9.3%). Summaries are available at LTLA level. Living Environment measures the quality of both indoor and outdoor local environments. The 'indoors' living environment measures the quality of housing; while the 'outdoors' living environment contains measures of air quality and road traffic accidents. [13]	https://www.gov.uk/government/statistics/english-indices-of-deprivation-2019	Patel et al [8], Bennett et al. [14], Webb, Blane, and de Vries [12], Pannullo et al. [7]	LTLA IMD score for Living Environment Deprivation Domain	static	IMD
			LTLA IMD score for Crime Domain	static	IMD
			LTLA IMD score for Barriers to Housing and Services Domain	static	IMD
			Population weighted average of the combined IMD ranks for the LSOAs in the LTLA	static	IMD
			Population weighted avg of the combined IMD overall score for the LSOAs in the area of interest	static	IMD
			Extent of deprivation within a local authority	static	IMD
			Concentration of deprivation within a local authority	static	IMD
2019 mid year population estimates based on the	https://www.nomisweb.co.u	OECD/European Union.	Population aged 16 to 24 in an area	static	age

ONS census available at LTLA level and above.	k/datasets/pestsyoala	"Mortality from respiratory diseases." Health at a glance: Europe 2018: state of health in the EU cycle [11]	Population aged 25 to 49 in an area	static	age
			Population aged 50 to 64 in an area	static	age
			Population aged over 64 in an area	static	age
			Population density for the LTLA (people/square km)	static	demo
			Percent of LTLA that are male	static	demo
			Percent of LTLA that are female	static	demo
Housing age data from the Valuation Office Agency<br>2020	https://data.cdrc.ac.uk/dataset/dwelling-ages-and-prices/resource/dwelling-age-group-counts-lsoa	Pevalin, Taylor, and Todd [9], Ellaway and Macintyre [2].	Percent of houses in LTLA built prior to 1919	static	housing
			Percent of houses in LTLA built prior to 1940	static	housing
			Percent of houses in LTLA built prior to 1973	static	housing
			Percent of houses in LTLA built prior to 1983	static	housing
Land use in England from 2018 live tables from the Department for Levelling Up, Housing and Communities and Ministry of Housing, Communities & Local Government	https://www.gov.uk/government/statistical-data-sets/live-tables-on-land-use	Gartner et al. [3]	Percent of land use associated with community buildings	static	land_use
			Percent of industrial land use in LTLA	static	land_use
			Percent of residential land use in LTLA	static	land_use
			Percent of land used by transport and utilities in LTLA	static	land_use
			Percent of agricultural land use in LTLA	static	land_use
			Percent of natural land use in LTLA	static	land_use
			Percent of land used by outdoor recreation (e.g. sports fields and parks) in LTLA	static	land_use
Property type/ethnicity/household composition	https://www.nomisweb.co.u	Prats-Uribe, Paredes and	Percent of people in LTLA of non white ethnicity	static	demo

ONS 2011 census datasets	k/sources/census_2011	Prieto-Alhambra [10]	Percentage of lone parent families in LTLA	static	demo
			Percent of "other children" in families in LTLA	static	demo
			Percent of detached houses in LTLA	static	housing
			Percent of semi-detached houses in LTLA	static	housing
			Percent of terraced houses in LTLA	static	housing
			Percent of flats in LTLA	static	housing
Weather. ERA5 data from European Centre for Medium-Range Weather Forecast	https://copernicus.eu/	Moriyama, Hugentobler & Iwasaki [5], Hajat, Bird & Haines [4], Nichols et al. [6]	Weekly average rainfall in LTLA	dynamic	weather
			Weekly total rainfall in LTLA	dynamic	weather
			Weekly minimum temperature in LTLA	dynamic	weather
			Weekly average temperature in LTLA	dynamic	weather
			Weekly maximum temperature in LTLA	dynamic	weather

Supplementary Table 3

Supplementary Table 3. Results from experimental models predicting registered deaths from respiratory disease 17 days in advance using over 2 billion in-store sales transactions with store location from March 2016 to March 2020.

Model	Months of the year included in results	Results on training set (approx. 70%) for 314 LTLAs			Results on test set (approx. 30%) for 314 LTLAs		
Additional models for further understanding and reference. Predicting Deaths in English Local Authorities 18/03/2016 to 27/03/2020		RMSE	MAE	R^2	RMSE	MAE	R^2
Random Forest Regressor with all variable group inputs apart from weather[a]	All months	2.8	2.03	0.85	3.41	2.38	0.79
Random Forest Regressor with all variable group inputs apart from IMD[a]	All months	2.55	1.88	0.88	3.44	2.40	0.78
Random Forest Regressor with all variable groups inputs apart from housing[a]	All months	2.55	1.88	0.88	3.43	2.39	0.78
Random Forest Regressor with all variable groups inputs apart from land use[a]	All months	2.53	1.87	0.88	3.43	2.39	0.78
Random Forest Regressor with all variable groups inputs apart from demographics (demographics variable group did not include age)[a]	All months	2.57	1.89	0.88	3.43	2.39	0.78
Additional Baseline Random Forest Regressor with input variables week number and LTLA identifier only	All months	3.21	2.29	0.81	4.21	2.93	0.67
Random Forest Regressor with all 56 variables[a] with the additional variable of LTLA identifier	All months	2.57	1.89	0.89	3.43	2.39	0.78

[a] All variables listed with description in supplement 4, including variable group names given in column 'Group for MCR'

Supplementary Table 4

Supplementary Table 4. Weekly prediction scores for PADRUS and PADRUNOS models

**Week Date**	PADRUS r2	PADRUS rmse	PADRUS mae	PADRUNOS r2 rank	PADRUNOS rmse	PADRUNOS mae
3/18/16	0.923143703	2.326358083	1.866016482	0.89011193	2.78171164	2.164473923
3/25/16	0.89580517	2.649106111	2.06727992	0.890912709	2.710586975	2.099802177
4/1/16	0.862391466	3.057716708	2.325069565	0.854028224	3.14926357	2.365569798
4/8/16	0.905041653	2.616247885	2.024457033	0.895170961	2.748863151	2.09863268
4/15/16	0.882198454	2.78265664	2.112326427	0.858623919	3.048400997	2.264835306
4/22/16	0.8461763	2.949696653	2.171230511	0.824293603	3.152532087	2.255535902
4/29/16	0.881210124	2.407228161	1.805160087	0.876087596	2.458583323	1.850121043
5/6/16	0.83161625	2.719577857	1.99325252	0.822192525	2.794643502	2.083724136
5/13/16	0.860779119	2.499133773	1.894076353	0.830121795	2.760616929	2.156522832
5/20/16	0.806148309	2.669376229	1.966955615	0.797478203	2.728417823	2.012581574
5/27/16	0.851137527	2.27224386	1.692685888	0.832646852	2.409235572	1.764447127
6/3/16	0.849032067	2.278846225	1.723427026	0.836768583	2.36959727	1.788301056
6/10/16	0.825700493	2.472617043	1.808580912	0.822171462	2.497523071	1.825285348
6/17/16	0.814209266	2.695904445	1.999641025	0.809008557	2.7333763	2.012720876

Continued on next page

Table 1 – continued from previous page

Week Date	PADRUS r2	PADRUS rmse	PADRUS mae	PADRUNOS r2 rank	PADRUNOS rmse	PADRUNOS mae
6/24/16	0.79976952	2.472324197	1.844448805	0.790128425	2.53114565	1.86692827
7/1/16	0.828777335	2.324693754	1.69289413	0.820272811	2.38172718	1.719078033
7/8/16	0.815231155	2.092012811	1.523230816	0.800164032	2.175638806	1.562943792
7/15/16	0.855849861	2.253742818	1.630632833	0.858736504	2.231062813	1.623640958
7/22/16	0.834360253	2.34001871	1.718712377	0.821095179	2.431913211	1.751051527
7/29/16	0.81892041	2.896613742	1.986478411	0.801708331	3.031154478	2.048624471
8/5/16	0.812869039	2.260216964	1.663356105	0.801806514	2.32606579	1.682749272
8/12/16	0.820454114	2.264787467	1.601356133	0.808061233	2.341645101	1.63596785
8/19/16	0.842251659	2.233354452	1.599234151	0.838741092	2.258068499	1.616994154
8/26/16	0.820283087	2.430019645	1.768889131	0.804456864	2.534758555	1.834687163
9/2/16	0.811190917	2.497957957	1.799506612	0.795320715	2.600821992	1.857927536
9/9/16	0.795699109	2.320430674	1.686573937	0.773637454	2.442506634	1.789300518
9/16/16	0.823375151	2.277536325	1.717619283	0.805949615	2.387243201	1.769905707
9/23/16	0.839614182	2.221858717	1.656336772	0.824574737	2.323697153	1.71712498
9/30/16	0.829258752	2.166738893	1.569475774	0.812022414	2.273476071	1.621753473
10/7/16	0.841668129	2.33000065	1.746458082	0.833881169	2.386609158	1.760064393
10/14/16	0.86875249	2.227992427	1.63896619	0.851991434	2.365982889	1.699400884
10/21/16	0.80659165	2.624017439	2.002397903	0.79564354	2.697262981	1.97696523
10/28/16	0.845621527	2.528366705	1.854892596	0.843724883	2.543850641	1.839958969
11/4/16	0.881835766	2.313472651	1.787106242	0.870520366	2.421709695	1.865435833
11/11/16	0.870646237	2.536810998	1.831885417	0.860590969	2.633564949	1.895349004
11/18/16	0.847695962	2.914849197	2.111086193	0.811305297	3.244443662	2.261632674
11/25/16	0.89276873	2.518300496	1.974261762	0.857958474	2.898374438	2.265943013
12/2/16	0.870362217	2.669616107	1.998052756	0.849905585	2.872534693	2.078132645

Continued on next page

Table 1 – continued from previous page

Week Date	PADRUS r2	PADRUS rmse	PADRUS mae	PADRUNOS r2 rank	PADRUNOS rmse	PADRUNOS mae
12/9/16	0.879381277	2.750067413	2.166490084	0.877449963	2.771996644	2.175278365
12/16/16	0.889793675	2.650602681	2.088106807	0.888074874	2.671192392	2.084830896
12/23/16	0.867022857	3.042788619	2.370230087	0.860389023	3.117762774	2.387752248
12/30/16	0.8766489	3.012103944	2.345668586	0.78219376	4.002520823	3.040899876
1/6/17	-11.00537055	7.877712295	6.834904906	-10.66828935	7.766331537	6.632395359
1/13/17	0.876308032	3.80833825	2.949707719	0.812430403	4.689710007	3.605574011
1/20/17	0.879155943	3.799830917	2.947332248	0.828006231	4.533232358	3.460603773
1/27/17	0.891972974	3.313322124	2.571206804	0.867383169	3.671104729	2.770057874
2/3/17	0.878935806	3.433160445	2.646682937	0.87014594	3.555609102	2.739185006
2/10/17	0.881905258	3.310537999	2.560122185	0.884631415	3.272103851	2.542940664
2/17/17	0.88590099	3.052421736	2.370306856	0.874266252	3.204273151	2.499951923
2/24/17	0.870761936	3.084772332	2.361804318	0.860487431	3.20504818	2.444548048
3/3/17	0.869452871	2.957764299	2.363514296	0.826550235	3.409312084	2.727518741
3/10/17	0.861219102	2.682868732	2.048442684	0.865060035	2.645482391	1.973987261
3/17/17	0.871278911	2.629875299	2.012583625	0.855378333	2.787577717	2.178360179
3/24/17	0.808736449	2.749214944	2.06360428	0.764405672	3.051230412	2.264829544
3/31/17	0.839522847	2.499677805	1.878847893	0.837867599	2.512536246	1.873175027
4/7/17	0.823701404	2.618867511	1.985868846	0.810029959	2.718514535	2.10046261
4/14/17	0.835277968	2.632012348	1.964910089	0.831272278	2.663822597	1.998059009
4/21/17	0.852006126	2.62347255	1.880461061	0.850943305	2.632875967	1.917931227
4/28/17	0.852698615	2.479218353	1.812715034	0.849069835	2.509570421	1.869533728
5/5/17	0.861357299	2.528380369	1.897316541	0.855052389	2.585231478	1.942613997
5/12/17	0.82382339	2.610394711	1.919444531	0.824218561	2.607465457	1.955959462
5/19/17	0.840550016	2.457747127	1.762576124	0.835485573	2.496473451	1.829649622

Continued on next page

Table 1 – continued from previous page

Week Date	PADRUS r2	PADRUS rmse	PADRUS mae	PADRUNOS r2 rank	PADRUNOS rmse	PADRUNOS mae
5/26/17	0.837806047	2.7215969	2.007810907	0.853953993	2.58256533	1.95798156
6/2/17	0.841750265	2.474924875	1.866478789	0.841231948	2.478974629	1.844219229
6/9/17	0.835148418	2.32920187	1.731451121	0.822486589	2.416997247	1.781764096
6/16/17	0.788875446	2.353760093	1.659289469	0.788107582	2.358036545	1.661075865
6/23/17	0.851379506	2.311939011	1.703330475	0.85323316	2.297476063	1.72185486
6/30/17	0.820129836	2.646518094	1.897242832	0.811074499	2.712317917	1.943954992
7/7/17	0.848307242	2.062688832	1.531419026	0.835774067	2.14620975	1.591996518
7/14/17	0.86456971	2.098647719	1.517111187	0.851837558	2.195081776	1.576864064
7/21/17	0.813056829	2.212475033	1.563924993	0.801776282	2.27825003	1.617234288
7/28/17	0.832221961	2.138525775	1.571932322	0.822002964	2.202689676	1.617549729
8/4/17	0.853265404	2.208469124	1.645340625	0.84563062	2.265195297	1.680907603
8/11/17	0.80777944	2.379013224	1.686452725	0.801668037	2.416536126	1.700495624
8/18/17	0.807259859	2.322960882	1.711190045	0.79776391	2.379496885	1.737513761
8/25/17	0.794568944	2.237922171	1.685590744	0.790422671	2.260393658	1.698786419
9/1/17	0.834703496	2.285251484	1.722957954	0.830514669	2.314025946	1.739723663
9/8/17	0.813723264	2.279504251	1.725071661	0.794886836	2.391981763	1.779478697
9/15/17	0.82909368	2.345851332	1.660133824	0.821181651	2.399537073	1.694508723
9/22/17	0.843761703	2.197431949	1.630958596	0.831859685	2.279594579	1.668860447
9/29/17	0.833377159	2.60345801	1.842209953	0.830744225	2.623946997	1.836998058
10/6/17	0.836354081	2.506780042	1.844226086	0.827212729	2.575843733	1.853430721
10/13/17	0.867201651	2.338101872	1.711885697	0.859914578	2.401394786	1.723054022
10/20/17	0.841199413	2.562787595	1.873413776	0.835754115	2.606356447	1.840921
10/27/17	0.864647302	2.318290833	1.721113019	0.846809387	2.466326314	1.777439692
11/3/17	0.850864739	2.72889911	2.008348029	0.84439798	2.787436143	1.939459882

Continued on next page

Table 1 – continued from previous page

Week Date	PADRUS r2	PADRUS rmse	PADRUS mae	PADRUNOS r2 rank	PADRUNOS rmse	PADRUNOS mae
11/10/17	0.869365959	2.544267785	1.868611636	0.858237338	2.650425301	1.905084118
11/17/17	0.835471811	2.756853733	2.068921006	0.82798584	2.818874012	2.090447157
11/24/17	0.828804518	2.757830921	2.131682977	0.825827087	2.78170963	2.129453684
12/1/17	0.876634627	2.719151419	2.061804128	0.868171668	2.810872408	2.069544449
12/8/17	0.858701597	2.833154467	2.204188736	0.837168477	3.041381285	2.273533942
12/15/17	0.873521068	3.057214894	2.377934123	0.864266918	3.167085066	2.45590052
12/22/17	0.861959759	3.318962088	2.451505324	0.851300667	3.444720156	2.553958123
12/29/17	0.891477303	3.230627369	2.479028947	0.870792356	3.525093844	2.690103898
1/5/18	0.85015842	4.980247341	3.776314851	0.816912795	5.505081309	4.147998893
1/12/18	0.892607552	3.962255076	2.984271803	0.78858718	5.559314203	4.244865305
1/19/18	0.895769315	3.75020512	2.927926283	0.838877529	4.662676476	3.529142052
1/26/18	0.886444503	3.611125699	2.777528138	0.852927897	4.109639014	3.142071327
2/2/18	0.886281992	3.381783385	2.686660451	0.88231172	3.440311541	2.767585353
2/9/18	0.885591593	3.097897859	2.384328182	0.887386245	3.073504444	2.431118047
2/16/18	0.839759924	4.135901566	2.932908911	0.875598326	3.644162506	2.690815404
2/23/18	0.881951415	3.24499937	2.474581367	0.886451273	3.182550915	2.478072049
3/2/18	0.876903059	3.406118311	2.500264615	0.885485026	3.285241038	2.520159945
3/9/18	0.84034371	3.943120785	2.99208176	0.868445069	3.579320847	2.761606978
3/16/18	0.885862913	3.09608043	2.393231871	0.894061313	2.982813738	2.347567051
3/23/18	0.862366043	3.119427278	2.333822671	0.860985908	3.135028422	2.329451525
3/30/18	0.82240037	3.328369087	2.458652501	0.832608904	3.231295143	2.441260955
4/6/18	0.894723415	2.551793438	2.000785804	0.864778144	2.892031833	2.169120534
4/13/18	0.867966796	2.567905687	1.923447216	0.87510862	2.497489782	1.924595531
4/20/18	0.830314112	2.520288368	1.977157204	0.797722408	2.751701115	2.19043934

Continued on next page

Table 1 – continued from previous page

Week Date	PADRUS r2	PADRUS rmse	PADRUS mae	PADRUNOS r2 rank	PADRUNOS rmse	PADRUNOS mae
4/27/18	0.817734013	2.52583929	1.927613468	0.798811936	2.653713323	2.033560904
5/4/18	0.804460249	2.627380589	1.901318424	0.783992481	2.761467218	2.02563923
5/11/18	0.809196082	2.487341307	1.823877722	0.800131076	2.545741929	1.850535691
5/18/18	0.808384392	2.3435427	1.764837756	0.711946488	2.8733863	2.12902062
5/25/18	0.829409277	2.408092561	1.719745766	0.826898402	2.425749832	1.734334745
6/1/18	0.811288064	2.410845263	1.786100343	0.794943041	2.513083436	1.852563491
6/8/18	0.839291326	2.445504265	1.738215626	0.834230928	2.483707894	1.764711571
6/15/18	0.852503318	2.155978743	1.599789663	0.852127465	2.158723942	1.612712969
6/22/18	0.815000978	2.278719145	1.649828196	0.792938705	2.410768721	1.728143668
6/29/18	0.835868311	2.224958619	1.609640904	0.829920372	2.264914788	1.69591454
7/6/18	0.81905667	2.421768314	1.633107582	0.809065834	2.487729334	1.680249076
7/13/18	0.866877337	2.033405412	1.509012902	0.856354993	2.112239916	1.556605428
7/20/18	0.834424458	2.252558125	1.573779246	0.831280023	2.273846627	1.60808512
7/27/18	0.789167319	2.336856685	1.751901879	0.765867248	2.462601829	1.8389052
8/3/18	0.875669718	1.806709237	1.389334716	0.862100841	1.90274511	1.453295848
8/10/18	0.80455667	2.122804574	1.580590261	0.783303999	2.235244506	1.657592582
8/17/18	0.815518535	2.054963131	1.501000953	0.782762671	2.229948803	1.620195441
8/24/18	0.834794782	1.951143301	1.489741593	0.812243749	2.080053423	1.600489133
8/31/18	0.826326725	2.312253479	1.621517707	0.807960233	2.431445446	1.713199836
9/7/18	0.843493741	1.955612159	1.484507519	0.826315348	2.060144084	1.554214922
9/14/18	0.810454429	2.134880765	1.580079261	0.777985305	2.310508999	1.685032067
9/21/18	0.833262068	2.327752451	1.711015485	0.826194188	2.376576154	1.726171398
9/28/18	0.774806625	2.502640292	1.782147903	0.764335906	2.560161349	1.8074722
10/5/18	0.83007698	2.188357678	1.692186237	0.837026569	2.143140296	1.644513912

Continued on next page

Table 1 – continued from previous page

Week Date	PADRUS r2	PADRUS rmse	PADRUS mae	PADRUNOS r2 rank	PADRUNOS rmse	PADRUNOS mae
10/12/18	0.86961965	2.276639418	1.754866066	0.86548391	2.312465689	1.750665568
10/19/18	0.796617802	2.536693074	1.948263583	0.804724243	2.485625063	1.859824013
10/26/18	0.773978114	2.552653928	1.886750324	0.805042765	2.370753338	1.71855966
11/2/18	0.81971565	2.579214482	1.927211825	0.820388386	2.574397777	1.856498478
11/9/18	0.830856267	2.620077356	1.942639863	0.831379026	2.616025388	1.933293502
11/16/18	0.731483596	2.960567933	2.251409234	0.674808136	3.258062831	2.450034592
11/23/18	0.846294725	2.550951783	1.915977074	0.84495641	2.562033308	1.933895292
11/30/18	0.85292012	2.408293666	1.790802612	0.851681524	2.418412821	1.767959639
12/7/18	0.872061129	2.521187324	1.912641647	0.86350214	2.604154697	1.99057639
12/14/18	0.874749597	2.719093216	2.154909762	0.86545322	2.818195935	2.202245735
12/21/18	0.894609194	2.555356825	1.9512748	0.879243842	2.735299158	2.028080686
12/28/18	0.887266931	2.74141673	2.180290567	0.870800455	2.934809293	2.234601264
1/4/19	0.67317327	4.366841361	3.439943959	0.589667413	4.89301599	3.68653107
1/11/19	0.836512482	3.825858103	3.069120368	0.848796328	3.679321406	2.815546174
1/18/19	0.799749944	3.997121393	3.029358283	0.819113157	3.798958111	2.849730462
1/25/19	0.726388299	4.450428841	3.420463115	0.771097083	4.070616534	3.129793778
2/1/19	0.850366596	3.495837066	2.49712213	0.827777333	3.750438601	2.647299698
2/8/19	0.83432607	3.574782932	2.686626817	0.813589402	3.791908834	2.888699834
2/15/19	0.818800548	3.587965624	2.785027235	0.79063681	3.856736757	2.989450335
2/22/19	0.805858345	3.599143229	2.811499652	0.759121397	4.009026044	3.074262544
3/1/19	0.853717651	2.986813881	2.334905696	0.826304977	3.254661937	2.541621973
3/8/19	0.758290233	3.402906087	2.445071073	0.763166149	3.368408476	2.439574627
3/15/19	0.803904977	3.250601533	2.367033784	0.803324576	3.255408536	2.35965737
3/22/19	0.81592342	2.760431789	2.08463928	0.799833181	2.87855012	2.143737333

Continued on next page

Table 1 – continued from previous page

Week Date	PADRUS r2	PADRUS rmse	PADRUS mae	PADRUNOS r2 rank	PADRUNOS rmse	PADRUNOS mae
3/29/19	0.749546864	3.292110477	2.436836491	0.65065937	3.888084666	2.862309006
4/5/19	0.773615515	3.18040092	2.307441769	0.760507731	3.271178848	2.369510306
4/12/19	0.781180282	3.091081793	2.309069402	0.783166283	3.077022534	2.320960727
4/19/19	0.767090671	3.227191825	2.370283671	0.754335164	3.314384104	2.434018397
4/26/19	0.804922248	3.237199079	2.265243328	0.815527829	3.147972768	2.221896455
5/3/19	0.844894192	2.562171583	1.892856881	0.835922833	2.63522835	1.967848814
5/10/19	0.800187002	2.660939689	1.961771709	0.791159697	2.720384682	1.975155606
5/17/19	0.791981007	2.71724395	1.991792252	0.77447177	2.829290829	2.095790122
5/24/19	0.789985644	2.737489046	1.948472203	0.757244803	2.943148437	2.095575218
5/31/19	0.782727888	2.794989753	1.980157676	0.771141433	2.868545966	2.017494721
6/7/19	0.805619768	2.508336094	1.845635856	0.789821689	2.608276502	1.881614161
6/14/19	0.779325557	2.652461127	1.898381908	0.784998136	2.618147566	1.875657971
6/21/19	0.79433644	2.469384772	1.744101344	0.774457624	2.585974071	1.785775456
6/28/19	0.785809879	2.680118961	1.940508892	0.771727384	2.766822258	2.01184751
7/5/19	0.781897979	2.523851898	1.842229026	0.765886043	2.614855378	1.875176513
7/12/19	0.774785002	2.470644799	1.800483224	0.777826984	2.453902555	1.76787506
7/19/19	0.778797543	2.428217529	1.688973657	0.784626643	2.396009887	1.686952105
7/26/19	0.770677326	2.612043811	1.887013635	0.776353463	2.579514936	1.872565487
8/2/19	0.787476424	2.439065228	1.786199007	0.782515261	2.467369837	1.810127738
8/9/19	0.750213101	2.525334775	1.75015423	0.74979012	2.52747203	1.742161801
8/16/19	0.692459954	2.608939971	1.821779209	0.683354459	2.647280361	1.837367855
8/23/19	0.715890235	2.769575549	1.903819338	0.702815838	2.832585098	1.934683078
8/30/19	0.787391529	2.455952638	1.738092876	0.788353157	2.450392204	1.778762536
9/6/19	0.721434907	2.525821686	1.820178221	0.686482135	2.679603199	1.921834493

Continued on next page

Table 1 – continued from previous page

Week Date	PADRUS r2	PADRUS rmse	PADRUS mae	PADRUNOS r2 rank	PADRUNOS rmse	PADRUNOS mae
9/13/19	0.80968294	2.575281255	1.800734894	0.804025687	2.613276614	1.836120715
9/20/19	0.804957307	2.481132537	1.752148959	0.806653208	2.470322235	1.748913566
9/27/19	0.770006987	2.705181158	1.905882137	0.773337637	2.685522149	1.901537565
10/4/19	0.783005613	2.814174806	2.016536542	0.783642759	2.810040232	1.939931591
10/11/19	0.743852541	3.452860216	2.449727162	0.722988571	3.590730535	2.474591217
10/18/19	0.779115929	2.782287255	2.022764333	0.788817121	2.720502448	1.976326271
10/25/19	0.724794921	3.33262644	2.300550428	0.722288501	3.347767924	2.267154748
11/1/19	0.848588556	2.727684173	2.040860919	0.817678389	2.993186709	2.178040935
11/8/19	0.838743749	2.92857178	2.103613862	0.816845252	3.121092821	2.141879116
11/15/19	0.809764723	3.3268415	2.464547197	0.798520326	3.42375124	2.49429345
11/22/19	0.833187869	2.978746901	2.182723187	0.812846337	3.155142324	2.230405621
11/29/19	0.782454498	3.284896773	2.503046633	0.73495435	3.625826456	2.692093717
12/6/19	0.833607335	3.32399506	2.575263412	0.829333877	3.366409482	2.573149076
12/13/19	0.830592406	3.642561475	2.66176968	0.802072841	3.937251417	2.785982017
12/20/19	0.812266504	3.96046277	2.876428077	0.815699449	3.924084661	2.833891815
12/27/19	0.794705079	4.256083064	3.271184809	0.760897241	4.593178114	3.39482626
1/3/20	0.480562326	5.077381199	3.929608995	0.351901169	5.671444247	4.225334707
1/10/20	0.780017301	4.073046639	2.988476469	0.794972796	3.932157134	2.933664609
1/17/20	0.810524858	3.520175978	2.695911592	0.787924954	3.72420037	2.755865219
1/24/20	0.802196643	3.517420542	2.608721089	0.786753713	3.652146632	2.623216079
1/31/20	0.778238402	3.535899136	2.644200711	0.712937532	4.022952488	2.972001221
2/7/20	0.782148836	3.437272756	2.679340199	0.591680217	4.705808656	3.556106374
2/14/20	0.739954532	3.664286494	2.791748679	0.583261135	4.638705272	3.458815905
2/21/20	0.852749188	2.848463246	2.269604197	0.813953966	3.201783623	2.431986167

Continued on next page

Table 1 – continued from previous page

Week Date	PADRUS r2	PADRUS rmse	PADRUS mae	PADRUNOS r2 rank	PADRUNOS rmse	PADRUNOS mae
2/28/20	0.801649489	3.329322885	2.470581364	0.659752735	4.360502861	3.230625194
3/6/20	0.790295617	3.268429614	2.493971463	0.752525543	3.550590386	2.743123341
3/13/20	0.791648362	3.323605875	2.420506913	0.743636306	3.686712906	2.662328426
3/20/20	0.732231527	4.78560102	3.123094724	0.722821421	4.868964397	3.173769107
3/27/20	0.500647333	9.404589524	5.410633635	0.387250167	10.41784181	6.138152103

Supplementary Table 5

Supplementary Table 5. Prediction scores by Lower Tier Local Authorities (LTLA) for PADRUS model including how many weeks (out of 211) where data reporting the deaths from respiratory disease may have been suppressed

ltla_code	ltla_name	No. of weeks with possible data suppression	r2	rmse	mae
E07000133	Melton	181	0.111770674	1.36979586	0.629941306
E07000166	Richmondshire	181	0.139800133	1.420526052	0.776969124
E07000167	Ryedale	176	0.15949007	1.398038935	0.738345912
E07000047	West Devon	175	0.205618021	1.233942928	0.613259061
E07000135	Oadby and Wigston	174	0.100169049	1.131934416	0.493256406
E06000017	Rutland	173	0.157321074	1.756780568	1.099428709
E07000124	Ribble Valley	170	0.173749956	1.26514735	0.612534121
E07000030	Eden	168	0.179703815	1.600306824	0.926341916
E07000163	Craven	167	0.166941579	1.197828965	0.648019173
E07000074	Maldon	165	0.254089743	1.455606282	0.65209883
E07000150	Corby	164	0.244531568	1.276666805	0.776068348
E07000029	Copeland	163	0.20577239	1.370972355	0.797784969
E07000125	Rossendale	159	0.190804891	1.094011733	0.749572195
E07000208	Epsom and Ewell	155	0.192699583	1.280467493	0.834715034

E07000042	Mid Devon	154	0.330951513	1.203113125	0.873755432
E07000218	North Warwickshire	152	0.249914639	1.32229467	0.803215806
E07000243	Stevenage	152	0.168889675	1.427396293	1.037236692
E07000169	Selby	151	0.138938648	1.461716982	0.996432967
E07000068	Brentwood	150	0.01490007	1.179033661	0.90957389
E07000136	Boston	150	0.203625197	1.210238849	0.862922721
E07000199	Tamworth	150	0.042447226	1.500987106	1.023059399
E07000156	Wellingborough	149	0.116028458	1.66707653	1.148691414
E07000151	Daventry	148	0.241345179	1.386482476	0.869541517
E07000077	Uttlesford	147	0.367704612	1.626897396	1.056236634
E07000083	Tewkesbury	147	0.066609737	1.080136121	0.793711193
E07000092	Rushmoor	147	0.171271788	1.774098492	1.190422753
E07000131	Harborough	147	0.199161365	1.359516707	0.814995938
E07000009	East Cambridgeshire	145	0.167451658	1.660993747	1.027929047
E07000223	Adur	145	0.338528828	1.317351647	0.898327087
E07000155	South Northamptonshire	144	0.261449899	1.499036492	0.919985297
E07000035	Derbyshire Dales	143	0.299506745	1.39008539	0.843327092
E07000103	Watford	139	0.241841001	1.32205428	0.977347593
E07000212	Runnymede	139	0.258782243	1.6150049	1.232544967
E07000129	Blaby	138	0.270413547	1.396126476	1.054942887
E09000020	Kensington and Chelsea	138	0.417827264	1.215771531	0.926348705
E07000027	Barrow-in-Furness	137	0.271644305	1.439741177	0.995614703
E07000089	Hart	137	0.15645297	1.612492486	1.152973108
E07000236	Redditch	137	0.177512764	1.549297597	1.05156235
E07000046	Torridge	135	0.255167921	1.252172161	0.816687648
E06000036	Bracknell Forest	134	0.038198823	1.33551174	1.0632991
E07000226	Crawley	131	0.29860962	1.767244996	1.202313896

E07000214	Surrey Heath	128	0.361875027	1.788141597	1.214257993
E07000044	South Hams	127	0.278735905	1.478886677	1.053701112
E07000079	Cotswold	127	0.183381259	1.603085107	1.169627441
E07000138	Lincoln	125	0.312356784	1.707478656	1.2992214
E07000080	Forest of Dean	124	0.248577965	1.719153842	1.237914795
E07000088	Gosport	124	0.400994357	1.493215136	1.034264219
E07000102	Three Rivers	123	0.19587674	1.524102563	1.119549462
E07000164	Hambleton	123	0.106991688	1.465141114	1.084582197
E07000095	Broxbourne	122	0.244277031	1.548613696	1.174903466
E07000008	Cambridge	121	0.200473108	1.73859678	1.292441607
E07000120	Hyndburn	120	0.268710311	1.483809651	1.086955179
E07000217	Woking	120	0.294594581	1.689560643	1.252543204
E07000142	West Lindsey	119	0.260339429	1.563055441	1.201128608
E07000075	Rochford	118	0.38360584	2.059114109	1.343016602
E06000039	Slough	117	0.257686078	1.973837307	1.519313734
E07000215	Tandridge	117	0.266933112	1.96646893	1.428987102
E07000134	North West Leicestershire	114	0.310326233	1.568695292	1.206785519
E07000073	Harlow	113	0.347683912	1.468421012	1.108272789
E07000107	Dartford	113	0.348159341	1.81053597	1.401947922
E07000109	Gravesham	112	0.318684887	1.8583586	1.443781449
E07000122	Pendle	111	0.337614232	1.546326892	1.146090508
E07000176	Rushcliffe	110	0.301116716	1.915798732	1.470977087
E07000203	Mid Suffolk	110	0.222401995	1.809894285	1.393970301
E07000241	Welwyn Hatfield	109	0.350614085	2.050375479	1.604984981
E07000220	Rugby	108	0.207047211	1.973541324	1.486759271
E07000153	Kettering	107	0.343206729	1.380204866	1.075113618
E07000098	Hertsmere	106	0.243635713	2.089094787	1.564433015

E07000210	Mole Valley	106	0.309017131	1.607832061	1.24342234
E07000213	Spelthorne	106	0.3632888	2.072321136	1.59736303
E07000033	Bolsover	105	0.283470032	1.865419256	1.350821346
E07000039	South Derbyshire	105	0.252932547	1.703294576	1.264782935
E07000132	Hinckley and Bosworth	102	0.147871492	1.739466525	1.377768116
E07000152	East Northamptonshire	102	0.311462769	1.621094459	1.206735459
E07000178	Oxford	102	0.176761154	2.084323378	1.62444658
E07000031	South Lakeland	101	0.233852538	1.75707297	1.454419484
E07000117	Burnley	101	0.433433611	1.839018443	1.387783395
E07000237	Worcester	101	0.297134113	1.75326371	1.368635926
E07000037	High Peak	100	0.295604359	1.922823699	1.42850402
E07000140	South Holland	100	0.357694387	1.932027672	1.375965406
E07000062	Hastings	99	0.33771796	1.679289014	1.33475864
E07000200	Babergh	97	0.241299177	1.541685805	1.170842883
E07000041	Exeter	96	0.328711648	1.902861532	1.49672537
E07000187	Mendip	96	0.332980709	1.858360893	1.557986973
E07000192	Cannock Chase	95	0.348657378	1.721018267	1.346404635
E07000043	North Devon	92	0.328921351	1.913289835	1.468134812
E07000119	Fylde	92	0.320086511	1.957873608	1.443141543
E09000013	Hammersmith and Fulham	92	0.483188698	1.658960121	1.324688376
E07000078	Cheltenham	91	0.303590267	2.162859569	1.603497967
E07000180	Vale of White Horse	91	0.434575853	1.984424864	1.641766527
E07000086	Eastleigh	90	0.283944484	2.08910452	1.608412736
E07000172	Broxtowe	90	0.312576807	2.077894886	1.717955479
E07000181	West Oxfordshire	90	0.369193206	1.977122522	1.527777547
E07000082	Stroud	88	0.287757742	2.24882207	1.733710377
E07000126	South Ribble	88	0.317145302	2.272792617	1.757514884

E07000026	Allerdale	87	0.260859089	2.282431595	1.856000912
E07000116	Tunbridge Wells	85	0.23633191	2.087390733	1.705795687
E07000118	Chorley	85	0.315629567	2.076154242	1.660058792
E07000093	Test Valley	84	0.169283918	2.251431223	1.745220564
E06000001	Hartlepool	83	0.396222753	1.910785664	1.400068698
E07000094	Winchester	83	0.315452371	2.13462609	1.702075083
E07000234	Bromsgrove	83	0.144698169	2.286163996	1.722072608
E09000012	Hackney	83	0.414963758	1.86780617	1.519064155
E07000087	Fareham	81	0.244746123	2.369153213	1.847833091
E07000081	Gloucester	80	0.339977482	1.934687972	1.5463343
E07000222	Warwick	80	0.204578125	2.162879527	1.821435213
E07000240	St Albans	80	0.348082385	2.147191218	1.755452343
E07000235	Malvern Hills	79	0.327277355	2.152960939	1.658565417
E07000139	North Kesteven	78	0.361563176	2.166630967	1.656343629
E07000196	South Staffordshire	78	0.34108054	2.605050727	1.977733375
E07000209	Guildford	78	0.323601835	2.144499762	1.623512341
E07000239	Wyre Forest	78	0.225443349	2.713754862	2.097769273
E07000111	Sevenoaks	77	0.326062837	2.255241361	1.795424382
E07000148	Norwich	77	0.400906139	2.121692568	1.733251443
E07000202	Ipswich	77	0.410605735	1.937139169	1.622573104
E09000019	Islington	77	0.467244358	2.046726631	1.650917397
E07000069	Castle Point	76	0.441125634	1.802389528	1.373818134
E07000115	Tonbridge and Malling	76	0.257278152	2.23121492	1.730540961
E07000188	Sedgemoor	76	0.320395291	2.021436275	1.605010707
E09000014	Haringey	76	0.430433871	1.831083745	1.583375716
E07000085	East Hampshire	75	0.175175568	2.095176345	1.693832949
E09000033	Westminster	74	0.398039453	1.805713378	1.514848606

E07000028	Carlisle	73	0.322649279	2.060365023	1.656011742
E07000036	Erewash	73	0.210801171	2.454397013	2.013339563
E07000194	Lichfield	72	0.379840862	2.124525808	1.740614549
E07000063	Lewes	70	0.337308458	2.114148475	1.686744008
E07000221	Stratford-on-Avon	70	0.353053766	2.219807989	1.827299118
E07000227	Horsham	70	0.272738167	2.435866997	1.98372167
E06000041	Wokingham	69	0.420735793	2.094811076	1.705820642
E07000099	North Hertfordshire	69	0.361983237	2.155639813	1.752920915
E06000005	Darlington	68	0.461192269	2.074411248	1.648024644
E07000064	Rother	68	0.496027022	2.53726494	1.923284379
E07000096	Dacorum	67	0.339639473	2.146205363	1.621119146
E07000105	Ashford	67	0.352930048	2.350361102	1.944142403
E07000175	Newark and Sherwood	67	0.336494136	2.358002683	1.843781886
E07000242	East Hertfordshire	67	0.247506407	2.156080902	1.737218785
E07000012	South Cambridgeshire	66	0.328111896	2.149309229	1.660124598
E07000173	Gedling	66	0.45790234	1.961765044	1.545067241
E07000207	Elmbridge	66	0.325453655	2.611789285	1.960334512
E07000216	Waverley	66	0.438385176	2.133823038	1.729442446
E07000238	Wychavon	65	0.354669156	2.475169719	2.052457483
E07000149	South Norfolk	64	0.281823075	2.544856417	2.071753718
E07000193	East Staffordshire	64	0.383209215	2.167809567	1.703087088
E07000198	Staffordshire Moorlands	64	0.382255083	2.611424433	2.000342587
E07000171	Bassetlaw	63	0.324178314	2.19022338	1.760767733
E09000007	Camden	62	0.373054153	2.189636112	1.748214857
E09000030	Tower Hamlets	62	0.377612686	2.173139331	1.61007194
E07000061	Eastbourne	61	0.429059837	2.425708947	1.997245149
E07000123	Preston	61	0.501765038	2.580810963	2.078884762

E07000177	Cherwell	61	0.191027805	2.34978176	1.895181387
E07000225	Chichester	61	0.426417876	2.362747804	1.882745361
E09000021	Kingston upon Thames	61	0.404106179	2.403191258	1.915196022
E07000229	Worthing	59	0.386473005	2.498721102	1.996549743
E06000037	West Berkshire	57	0.256364049	2.496393489	2.033410043
E07000084	Basingstoke and Deane	57	0.403223184	2.6648679	2.104470671
E09000002	Barking and Dagenham	57	0.449911365	2.296495916	1.911073329
E06000038	Reading	56	0.279641948	2.42263231	1.942101495
E07000034	Chesterfield	55	0.406974462	2.544161664	1.988765597
E07000127	West Lancashire	55	0.289610312	2.726657904	2.116502114
E07000174	Mansfield	55	0.364035658	2.320204828	1.785221993
E09000027	Richmond upon Thames	55	0.398440547	2.16876519	1.83718613
E07000010	Fenland	54	0.308499109	2.499457316	2.071320499
E07000145	Great Yarmouth	54	0.420382962	2.287433428	1.806083155
E07000071	Colchester	52	0.471389919	2.628635454	2.13596208
E07000179	South Oxfordshire	52	0.283962503	2.439187304	1.970030204
E07000038	North East Derbyshire	50	0.280908901	2.339242444	1.890812458
E07000090	Havant	49	0.367649637	2.743987356	2.194871731
E07000108	Dover	49	0.386197105	2.764554528	2.182179977
E07000228	Mid Sussex	49	0.451153793	2.46169994	1.926512374
E07000170	Ashfield	48	0.310734764	2.455020544	1.89087169
E07000197	Stafford	48	0.365960757	3.009887069	2.260162675
E07000219	Nuneaton and Bedworth	48	0.322531445	2.294739077	1.841036698
E09000024	Merton	48	0.5087476	2.153793758	1.749814618
E06000034	Thurrock	47	0.345579969	2.529251347	2.100398734
E06000002	Middlesbrough	45	0.521167788	2.388084264	1.988882055
E07000144	Broadland	45	0.396901961	2.730686158	2.122338426

E09000031	Waltham Forest	45	0.458662899	2.600700072	2.066692631
E07000032	Amber Valley	44	0.297088219	2.426062958	1.960007608
E07000195	Newcastle-under-Lyme	44	0.396102427	3.036404185	2.316043249
E09000028	Southwark	44	0.381679503	2.491732617	2.02840859
E06000040	Windsor and Maidenhead	43	0.383433863	2.660160335	2.050889994
E07000070	Chelmsford	43	0.366748115	2.840284819	2.253542394
E07000168	Scarborough	43	0.338487908	2.394431319	1.932733741
E06000055	Bedford	42	0.404674639	2.915079041	2.314752321
E07000130	Charnwood	42	0.384539284	2.450519146	1.957608506
E07000147	North Norfolk	42	0.349249415	2.56501646	2.092754001
E09000025	Newham	42	0.412839447	2.175997865	1.799741281
E09000001	City of London	41	0.593173179	1.288580513	1.006316848
E06000006	Halton	40	0.354741921	2.319544946	1.888176338
E07000128	Wyre	40	0.485927885	2.92096545	2.347182638
E06000008	Blackburn with Darwen	38	0.318882442	2.731338862	2.211835206
E07000067	Braintree	38	0.416990322	2.563882126	2.049383259
E07000072	Epping Forest	38	0.388167975	2.78116347	2.16632607
E07000211	Reigate and Banstead	37	0.480462649	2.806200091	2.200082788
E06000020	Telford and Wrekin	36	0.364726955	2.623548894	2.157624168
E07000112	Folkestone and Hythe	36	0.200252145	2.934396635	2.200763638
E07000246	Somerset West and Taunton	36	0.384806296	3.100305882	2.507507779
E07000141	South Kesteven	35	0.346808555	2.75627896	2.057274028
E07000121	Lancaster	33	0.478872214	2.486515084	1.927616489
E06000003	Redcar and Cleveland	32	0.527701422	2.678551006	2.048041621
E07000045	Teignbridge	31	0.449144368	2.760674532	2.163972118
E07000143	Breckland	31	0.438600989	2.917791453	2.262947315
E09000032	Wandsworth	31	0.406141598	2.726982864	2.263841329

E06000022	Bath and North East Somerset	29	0.415011234	2.359751178	1.920177313
E06000032	Luton	29	0.43537366	2.950250195	2.388267105
E09000022	Lambeth	29	0.452977297	2.68871032	2.066663573
E06000046	Isle of Wight	28	0.463663932	3.198368534	2.626490423
E07000011	Huntingdonshire	28	0.37303477	3.140086462	2.515344174
E07000113	Swale	28	0.359298545	2.623988643	2.11756356
E09000029	Sutton	28	0.318254856	3.167572072	2.420641234
E06000053	Isles of Scilly	27	0.579060613	1.013347961	0.607850407
E07000110	Maidstone	27	0.273701283	3.486349702	2.786984669
E09000015	Harrow	27	0.502291302	2.502274771	2.03164294
E07000165	Harrogate	26	0.45630331	2.938653919	2.205212978
E09000026	Redbridge	25	0.539770352	3.057460363	2.450946662
E07000040	East Devon	24	0.526858162	2.634455026	2.049187174
E07000189	South Somerset	24	0.407513996	3.088208951	2.204938982
E07000245	West Suffolk	24	0.468461745	2.829031827	2.103031699
E09000011	Greenwich	24	0.438425446	2.914507712	2.269327115
E06000014	York	21	0.561322588	3.131399462	2.385603713
E09000023	Lewisham	21	0.404747815	2.923714646	2.360343376
E08000011	Knowsley	20	0.430257815	3.181313441	2.560334288
E06000031	Peterborough	19	0.426214714	2.976000479	2.391185219
E07000065	Wealden	19	0.530701627	2.909721775	2.259201224
E07000106	Canterbury	19	0.446817878	2.974176131	2.317181125
E07000066	Basildon	18	0.471034447	2.999980642	2.410503667
E07000224	Arun	17	0.491401962	3.537358499	2.728049479
E06000027	Torbay	16	0.418177914	3.167924968	2.524796314
E07000146	King's Lynn and West Norfolk	16	0.351300685	3.218801963	2.638632304
E09000005	Brent	16	0.404896515	2.709185136	2.208130649

E09000018	Hounslow	16	0.421077365	2.966309821	2.444971853
E06000004	Stockton-on-Tees	15	0.425873527	3.463497061	2.633367162
E06000012	North East Lincolnshire	15	0.404652828	3.20824508	2.501206934
E06000013	North Lincolnshire	13	0.431899456	2.974122093	2.434753182
E07000154	Northampton	13	0.474248067	3.252684439	2.52520773
E06000042	Milton Keynes	12	0.466915259	3.056752864	2.453923403
E06000044	Portsmouth	12	0.490157434	3.161109445	2.545453086
E08000002	Bury	12	0.462037228	3.146634118	2.484383806
E06000033	Southend-on-Sea	11	0.519976639	3.77985657	2.953389889
E07000137	East Lindsey	11	0.498671134	3.024131478	2.407419159
E06000024	North Somerset	10	0.525052044	3.775220968	3.065330216
E07000114	Thanet	10	0.507305088	3.204776871	2.627700328
E09000009	Ealing	10	0.475910986	3.207512406	2.53797207
E06000030	Swindon	9	0.420935125	3.426253337	2.706470678
E06000043	Brighton and Hove	9	0.489243474	3.165270349	2.548342715
E08000023	South Tyneside	9	0.538734504	2.857885761	2.258325862
E08000029	Solihull	9	0.403287704	3.297619593	2.615964388
E08000033	Calderdale	8	0.489364788	3.004501171	2.366135244
E06000009	Blackpool	7	0.622048719	3.038791897	2.494436727
E06000019	Herefordshire, County of	7	0.513577734	3.118844378	2.501775531
E06000045	Southampton	7	0.553733797	3.30460847	2.679914324
E08000009	Trafford	7	0.390890592	3.422440261	2.680342325
E06000007	Warrington	6	0.42646624	3.111167671	2.422071489
E08000004	Oldham	6	0.407582416	3.74485268	2.912279027
E06000056	Central Bedfordshire	5	0.520767132	3.292944992	2.600599577
E08000005	Rochdale	5	0.561728887	3.032403191	2.356420897
E08000006	Salford	5	0.430393618	3.84259213	2.902171568

E08000022	North Tyneside	5	0.530638646	3.495884543	2.737176482
E09000016	Havering	5	0.519233128	3.70223238	2.817872162
E06000016	Leicester	4	0.544016443	3.730256615	2.929819298
E06000025	South Gloucestershire	4	0.503166465	3.479222836	2.707519081
E08000013	St. Helens	4	0.419507361	3.212971913	2.53512388
E08000016	Barnsley	4	0.667824427	3.364878777	2.716219963
E09000010	Enfield	4	0.519920053	2.948689751	2.305750097
E06000015	Derby	3	0.489558022	3.653966678	2.862052513
E06000018	Nottingham	3	0.550701854	3.610795696	2.918996624
E06000026	Plymouth	3	0.476965249	3.777626487	2.85838702
E07000076	Tendring	3	0.556662811	3.592406462	2.848978896
E07000091	New Forest	3	0.56520888	3.349563126	2.718663098
E08000008	Tameside	3	0.529346139	3.840109426	3.015948452
E08000018	Rotherham	3	0.511625194	4.17454424	3.286956802
E08000037	Gateshead	3	0.494410269	3.543818384	2.762522599
E09000017	Hillingdon	3	0.532608966	3.574185144	2.922425365
E06000035	Medway	2	0.492691009	4.029803221	3.176422708
E08000017	Doncaster	2	0.627368525	4.185811924	3.357865324
E08000026	Coventry	2	0.528344276	4.032848764	3.174099171
E09000004	Bexley	2	0.434397218	3.885308067	2.952734136
E09000006	Bromley	2	0.534756074	4.445095767	3.36872832
E06000021	Stoke-on-Trent	1	0.520243717	4.431107859	3.451877108
E06000023	Bristol, City of	1	0.673715056	3.759568567	2.882541908
E06000049	Cheshire East	1	0.665595954	4.875422952	3.772057145
E06000051	Shropshire	1	0.631782866	4.163053097	3.23002622
E06000052	Cornwall	1	0.805704951	5.404671002	4.225539956
E06000054	Wiltshire	1	0.701683235	4.429271026	3.447777407

E06000058	Bournemouth, Christchurch and Poole	1	0.754665268	4.205065198	3.211494353
E06000060	Buckinghamshire	1	0.736767361	4.451849362	3.524168221
E07000244	East Suffolk	1	0.64297824	3.872449949	3.029766225
E08000003	Manchester	1	0.737115317	3.74306494	2.870577499
E08000007	Stockport	1	0.475379439	3.90161322	3.053871215
E08000015	Wirral	1	0.623998932	4.347692765	3.384701146
E08000021	Newcastle upon Tyne	1	0.502096342	3.73949699	3.006697927
E08000025	Birmingham	1	0.889415265	4.603059486	3.617231396
E08000027	Dudley	1	0.550453491	4.735651382	3.791939012
E08000028	Sandwell	1	0.623705741	3.904817103	3.002966231
E08000030	Walsall	1	0.528130917	4.170909569	3.303256279
E08000031	Wolverhampton	1	0.532933426	4.111649094	3.160146861
E08000032	Bradford	1	0.709380337	4.230069963	3.31687929
E08000034	Kirklees	1	0.653884399	4.384541946	3.495201562
E09000003	Barnet	1	0.595804435	3.391800278	2.676771902
E09000008	Croydon	1	0.535777702	4.299938037	3.366876543
E06000010	Kingston upon Hull, City of	0	0.529587637	3.686492904	3.085802199
E06000011	East Riding of Yorkshire	0	0.7058961	4.185541837	3.15622795
E06000047	County Durham	0	0.848060712	4.323880831	3.317022017
E06000050	Cheshire West and Chester	0	0.62561863	3.883508244	3.026697108
E06000057	Northumberland	0	0.644938137	4.169249356	3.39783922
E06000059	Dorset	0	0.661155806	4.789258677	3.838467277
E08000001	Bolton	0	0.450865214	4.512825564	3.558860741
E08000010	Wigan	0	0.481865861	4.544990709	3.503837143
E08000012	Liverpool	0	0.763102749	4.503774875	3.471418707
E08000014	Sefton	0	0.664543348	4.032142747	3.095096912
E08000019	Sheffield	0	0.767299236	4.352941998	3.366375769

E08000024	Sunderland	0	0.522111995	4.134906828	3.241375668
E08000035	Leeds	0	0.804382085	5.4065491	4.043894947
E08000036	Wakefield	0	0.572919111	4.064629659	3.328229927
