# Supplementary material for: Assessing the value of integrating national longitudinal shopping data into respiratory disease forecasting models
Source: Nat Commun. 2023 Nov 21;14:7258. doi: 10.1038/s41467-023-42776-4 (PMC10663456; doi:10.1038/s41467-023-42776-4)
Supplement: Supplementary file 3 — Reporting Summary [file 41467_2023_42776_MOESM3_ESM.pdf]

nature portfolio

Corresponding author(s): Elizabeth Dolan

Last updated by author(s): Oct 6, 2023

Reporting Summary

Nature Portfolio wishes to improve the reproducibility of the work that we publish. This form provides structure for consistency and transparency in reporting. For further information on Nature Portfolio policies, see our Editorial Policies and the Editorial Policy Checklist.

Statistics

For all statistical analyses, confirm that the following items are present in the figure legend, table legend, main text, or Methods section.

n/a	Confirmed	
☐	☒	The exact sample size (*n*) for each experimental group/condition, given as a discrete number and unit of measurement
☐	☒	A statement on whether measurements were taken from distinct samples or whether the same sample was measured repeatedly
☐	☒	The statistical test(s) used AND whether they are one- or two-sided<br>*Only common tests should be described solely by name; describe more complex techniques in the Methods section.*
☐	☒	A description of all covariates tested
☐	☒	A description of any assumptions or corrections, such as tests of normality and adjustment for multiple comparisons
☐	☒	A full description of the statistical parameters including central tendency (e.g. means) or other basic estimates (e.g. regression coefficient) AND variation (e.g. standard deviation) or associated estimates of uncertainty (e.g. confidence intervals)
☐	☒	For null hypothesis testing, the test statistic (e.g. *F*, *t*, *r*) with confidence intervals, effect sizes, degrees of freedom and *P* value noted<br>*Give P values as exact values whenever suitable.*
☒	☐	For Bayesian analysis, information on the choice of priors and Markov chain Monte Carlo settings
☐	☒	For hierarchical and complex designs, identification of the appropriate level for tests and full reporting of outcomes
☒	☐	Estimates of effect sizes (e.g. Cohen's *d*, Pearson's *r*), indicating how they were calculated

Our web collection on statistics for biologists contains articles on many of the points above.

Software and code

Policy information about availability of computer code

Data collection	Open source PostgreSQL 13
Data analysis	Open-source software Python v.3.8 with the following python libraries were used in the analysis: Matplotlib 3.5.1, MCRForest (https://github.com/gavin-s-smith/mcrforest), Numpy 1.21.4, Pandas 1.3.5, Seaborn 0.11.2, Scikit-learn 0.24.2, SciPy 1.5.4, SHAP 0.39.0<br>Code deposition in a community repository: https://github.com/nhsx/commercial-data-healthcare-predictions

For manuscripts utilizing custom algorithms or software that are central to the research but not yet described in published literature, software must be made available to editors and reviewers. We strongly encourage code deposition in a community repository (e.g. GitHub). See the Nature Portfolio guidelines for submitting code & software for further information.

Data

Policy information about availability of data

All manuscripts must include a data availability statement. This statement should provide the following information, where applicable:

- Accession codes, unique identifiers, or web links for publicly available datasets
- A description of any restrictions on data availability
- For clinical datasets or third party data, please ensure that the statement adheres to our policy

The health data used in this study is not publicly available but can be requested via NHS England and Improvement NCDR. The shopping dataset used in this study is commercially sensitive and therefore not available for access. All other datasets are open source and can be accessed via the website links given in Supplement 1

[which are as follows: https://www.gov.uk/government/statistics/english-indices-of-deprivation-2019, https://www.nomisweb.co.uk/datasets/pestsyoala, https://data.cdrc.ac.uk/dataset/dwelling-ages-andprices/resource/dwellingage-group-counts-lsoa, https://www.gov.uk/government/statistical-data-sets/live-tables-on-land-use, https://www.nomisweb.co.uk/sources/census_2011, https://www.copernicus.eu/en].

Research involving human participants, their data, or biological material

Policy information about studies with human participants or human data. See also policy information about sex, gender (identity/presentation), and sexual orientation and race, ethnicity and racism.

Reporting on sex and gender	N/A
Reporting on race, ethnicity, or other socially relevant groupings	N/A
Population characteristics	N/A
Recruitment	N/A
Ethics oversight	N/A

Note that full information on the approval of the study protocol must also be provided in the manuscript.

Field-specific reporting

Please select the one below that is the best fit for your research. If you are not sure, read the appropriate sections before making your selection.

☐ Life sciences ☒ Behavioural & social sciences ☐ Ecological, evolutionary & environmental sciences

For a reference copy of the document with all sections, see nature.com/documents/nr-reporting-summary-flat.pdf

Behavioural & social sciences study design

All studies must disclose on these points even when the disclosure is negative.

Study description	The study investigated the value of integrating sales of non-prescription medications commonly bought for managing respiratory symptoms, to improve forecasting of weekly registered deaths from respiratory disease at local levels across England, by using over 2 billion transactions logged by a UK high street retailer from March 2016 to March 2020. The study reports the results from the novel AI explainability variable importance tool Model Class Reliance implemented on the PADRUS model. PADRUS is a machine learning model optimised to predict registered deaths from respiratory disease in 314 local authority areas across England through the integration of shopping sales data and focused on purchases of non-prescription medications. The data and study are quantitative.
Research sample	Data underpinning medication sales variables originates from two data-sets made available to the project by a national UK high street retailer, incorporating: 1. a 20% sample of timestamped commercial sales transactions (including over 5 million transactions of non-prescription medications for cough, decongestant and throat) from England and Wales recorded through 2,702,449 loyalty cards from November 2009 to April 2015; and 2. a data-set of all in-store weekly commercial sales transactions in England (over 2 billion) labelled by store location, covering the period between March 2016 to March 2020. Commercial sales data was sales units of all in-store transactions with store location only; no sales transactions were linked to individual customers, and no personal data was used in this study. Other independent variables used in the models are derived from: English indices of deprivation 2019 ; Nomis official census and labour market statistics; Housing age data from the Valuation Office Agency 2020; ONS Census 2011; Land use in England from 2018 live tables from the Department for Levelling Up, Housing and Communities and Ministry of Communities and Local Government; and Weather ERA5 data from the European Centre for Medium-Range Weather Forecasts. Data sources and descriptions used in this study can be seen in Supplement 1, which lists the data used for both dependent and independent variables. The output variable for the models, respiratory deaths in each of the England's 314 Local Authorities (LTLAs), originates from two datasets supplied by the UK Office of National Statistics (ONS) and the National Commissioning Data Repository (NDCR) containing: 1. details of total weekly registered deaths from respiratory disease (as the underlying cause)(ICD 10 coding: J00 - J99) in England and Wales from 7th December 2009 to 13th April 2015; and 2. ONS data on all weekly registered deaths from respiratory disease (ICD 10 coding: J00 - J99) by 314 LTLAs in England from 18th March 2016 to 27th March 2020. No sample size calculations were performed, however, the data was representative as 1) a randomized selection of 20% consumer data 2) all sales transactions for the whole of England, ensuring comprehensive coverage and representation. Data outside the consumer datasets were also considered representative, given their population-level coverage for the entirety of England. The sample size was determined by the fact that the retailer's consumer data encompassed the entire population of individuals who shopped there.
Sampling strategy	Sales dataset 1, for exploratory modelling, was a 20% sample of sales transactions recorded though loyalty cards. Sales dataset 2, used for the principle experiment, no sampling was used as all in-store transactions in England were used. These samples were determined to be sufficient as the first dataset was for exploratory analysis only but still captured sales from 2,702,449 people in the UK population, and the second dataset captured the sales of the entire population of customers shopping in-store at the retailer in England. No sample size calculations were performed, however, the data was representative as 1) a randomized selection of 20% consumer data 2) all sales transactions for the whole of England, ensuring comprehensive coverage and representation. Data outside the consumer datasets were also considered representative, given their population-level coverage for the entirety of England.

Data collection	Sales data was recorded and stored by a national UK high street retailer across the UK through point-of-sale logging systems in stores. This transactional shopping data consists of longitudinal, time-stamped purchasing logs, specified at store-level geographical granularity. Sales data was collected through two main sources: customers' loyalty cards and electronic till logs. Given that the study primarily focused on analysing purchasing patterns and did not involve traditional experimental conditions or hypotheses, the concept of blinding the researcher to experimental conditions or study hypotheses was not applicable to this research.
Timing	Sales dataset 1 used for exploratory analysis gives purchasing logs collected from 1st November 2009 to 13th April 2015. Sales dataset 2 used for the principle experiment gives purchasing logs collecting from 18th March 2016 to 27th March 2020. There were no gaps in data collection in either of these time periods.
Data exclusions	No data were excluded from the analysis.
Non-participation	Data was only used at store level, there were no participants and no individual customer information was known.
Randomization	No sales transactions were linked to individual customers, and no personal data was used in this study, therefore there were no experimental groups.

Reporting for specific materials, systems and methods

We require information from authors about some types of materials, experimental systems and methods used in many studies. Here, indicate whether each material, system or method listed is relevant to your study. If you are not sure if a list item applies to your research, read the appropriate section before selecting a response.

Materials & experimental systems

n/a	Involved in the study
☒	☐ Antibodies
☒	☐ Eukaryotic cell lines
☒	☐ Palaeontology and archaeology
☒	☐ Animals and other organisms
☒	☐ Clinical data
☒	☐ Dual use research of concern
☒	☐ Plants

Methods

n/a	Involved in the study
☒	☐ ChIP-seq
☒	☐ Flow cytometry
☒	☐ MRI-based neuroimaging
